# Patients’ and health care providers’ perspectives on quality of hemophilia care in the Netherlands: a questionnaire and interview study

**DOI:** 10.1016/j.rpth.2023.100159

**Published:** 2023-04-23

**Authors:** Martijn R. Brands, Lotte Haverman, Jelmer J. Muis, Mariëtte H.E. Driessens, Felix J.M. van der Meer, Geertje Goedhart, Stephan Meijer, Marianne de Jong, Johanna G. van der Bom, Marjon H. Cnossen, Karin Fijnvandraat, Samantha C. Gouw, HiN6 Steering Group, L.F.D. van Vulpen, J. Eikenboom, E.A.M. Beckers, L. Hooimeijer, P.F. Ypma, L. Nieuwenhuizen, M. Coppens, S.E.M. Schols, B.A.P. Laros, P.R. Valk, M.H. Cnossen, M.H.E. Driessens, J.G. van der Bom, F.R. Rosendaal, C. Smit, F.W.G. Leebeek, S.C. Gouw, S. Hassan, E.C. van Balen, J.G. van der Bom, C. Smit, F.R. Rosendaal, S.C. Gouw, SYMPHONY consortium@SYMPHONY_NL, M.H. Cnossen, S.H. Reitsma

**Affiliations:** 1 Department of Pediatric Hematology, Emma Children’s Hospital, Amsterdam University Medical Centers location University of Amsterdam, Meibergdreef 9, Amsterdam, the Netherlands; 2 Amsterdam Reproduction & Development, Public Health, Amsterdam, the Netherlands; 3 Department of Child and Adolescent Psychiatry & Psychosocial Care, Emma Children’s Hospital, Amsterdam University Medical Centers location University of Amsterdam, Meibergdreef 9, Amsterdam, the Netherlands; 4 Netherlands Hemophilia Patient Society, Nijkerk, the Netherlands; 5 HemoNED Foundation, Leiden, the Netherlands; 6 Department of Thrombosis and Hemostasis, Leiden University Medical Center, Leiden, the Netherlands; 7 BuroMD, Utrecht, the Netherlands; 8 Department of Clinical Epidemiology, Leiden University Medical Center, Leiden, the Netherlands; 9 Department of Pediatric Hematology, Sophia Children’s Hospital, Erasmus University Medical Center, Erasmus University Rotterdam, Rotterdam, the Netherlands; 10 Department of Molecular Cellular Hemostasis, Sanquin Research and Landsteiner Laboratory, Amsterdam, the Netherlands

**Keywords:** health policy, hemophilia A, hemophilia B, patient satisfaction, quality of health care, telemedicine

## Abstract

**Background:**

Hemophilia care has improved greatly because of advances in treatment options and comprehensive care. In-depth insight into the perspectives of persons with hemophilia and health care providers on their care may provide targets for further improvements.

**Objectives:**

To assess satisfaction of the hemophilia population with their care, to explore factors determining care satisfaction, and to identify areas for potential health care improvements, including digital health tools.

**Methods:**

First, to assess care satisfaction and factors determining satisfaction and health care improvements, data from a nationwide, cross-sectional questionnaire among 867 adult and pediatric Dutch persons with hemophilia A or B were analyzed. This included the Hemophilia Patient Satisfaction Scale questionnaire, Canadian Hemophilia Outcomes Kids’ Life Assessment Tool satisfaction questions, a visual analog scale satisfaction score, and open questions. Second, to further explore factors determining satisfaction and health care improvements, semistructured interviews were conducted with 19 persons with hemophilia or their parents and 18 health care providers.

**Results:**

High care satisfaction was found, with an overall median Hemophilia Patient Satisfaction Scale score of 12 (IQR, 6-21). Participants in the interviews reported that patient-professional interactions, availability of care, and coordination of care were major factors determining satisfaction. Suggested health care improvements included improved information provision and coordination of care, especially shared care with professionals not working within comprehensive care centers. Participants suggested that digital health tools could aid in this.

**Conclusion:**

Satisfaction with hemophilia care is high among persons with hemophilia in the Netherlands, although several potential improvements have been identified. Accentuating these is especially relevant in the current era of treatment innovations, in which we might focus less on other aspects of care.

## Essentials

• The perceived quality of care by people with hemophilia is determined by different elements.
• We assessed care satisfaction by conducting a nationwide questionnaire and in-depth interviews.
• Satisfaction was mainly determined by care availability, communication, and coordination of care.
• Desired improvements related to information provision, coordination responsibilities, and e-Health.

## 1 Introduction

Hemophilia is an inherited bleeding disorder caused by a deficiency of functional coagulation factor VIII (hemophilia A) or factor IX (hemophilia B). Three types of hemophilia are distinguished: severe hemophilia (defined as a residual clotting factor activity of less than 1%), moderate-severe hemophilia (between 1% and 5%), and mild hemophilia (between 6% and 40%). Severely affected patients have spontaneous joint and muscle bleedings leading to joint damage. As a consequence, life-long prophylactic treatment to prevent bleeds is needed. People regularly self-infuse with coagulation factor products or nonfactor replacement products. Such home treatment with remote advice from health care providers demands high self-management.

Hemophilia care improvements have predominantly been the result of major advances in treatment [1,2]. Additionally, the centralization of Dutch hemophilia care in 6 comprehensive care centers (CCCs) resulted in specialized, multidisciplinary care. Together, this led to improved life expectancy, decreased annual bleeding rate, and improved social participation [3,4].

Yet, people’s perception of the quality of their care is not determined by these health outcomes alone. Reported additional elements of care satisfaction are ease of treatment administration, burden of disease, relationships with health care providers, health care costs, information provision, and coordination of care [[5], [6], [7], [8], [9], [10]]. Coordination of care is defined as the organization of care activities between 2 or more participants involved in a patient’s care, including the patient himself/herself [11]. Additionally, the organization of a health care system is an important determinant of satisfaction [9]. The Dutch health care system is often ranked as one of the highest in the world, as illustrated in Box A [12,13]. Finally, recent advances in digital technology have changed the landscape of health care. As in other medical fields, the COVID-19 pandemic accelerated the use of digital health tools in hemophilia care.

Box A **Organization of Dutch health care.**

Due to a compulsory health insurance, which is provided by insurers acting in competition, the Dutch health care system is relatively efficient and accessible, as compared to others. [13] Coagulation factor costs are covered by public insurance, except from a yearly deductible fee of approximately €400, which is paid by individuals themselves. Children aged of <18 years are insured through their parents. For them, no additional fee is required.

In-depth insight into how people with hemophilia perceive the quality of Dutch hemophilia care may provide targets for further improvement. Therefore, in this study, we aimed to assess perspectives of the Dutch hemophilia population on the quality of their care. First, we assessed the degree of their satisfaction with hemophilia care. Second, we explored factors determining care satisfaction. Third, we identified areas for potential health care improvements, with a special focus on digital health tools. Patient perspectives were juxtaposed with health care provider perspectives to help determine which improvements are most desired.

## 2 Methods

This mixed methods study consisted of 2 parts. First, in a quantitative study of nationwide, cross-sectional questionnaire data, we assessed patient satisfaction with hemophilia care, factors determining care satisfaction, and areas for potential health care improvements. Second, in a qualitative study of semistructured interviews, we further explored the latter 2 topics: factors determining care satisfaction and potential health care improvements.

### 2.1 Quantitative study

This study was part of the sixth nationwide “Hemophilia in the Netherlands” (HiN6) study, the details of which are published elsewhere [3,4].

#### 2.1.1 Participants

From June 2018 until July 2019, all male pediatric and adult patients with severe, moderate-severe, and mild hemophilia A or B known in the Netherlands were invited to participate. Parents of children (0-11 years), adolescents (between 12 and 17 years), and adults (18 years and older) completed age-specific questionnaires.

#### 2.1.2 Measures

**Figure 1:**
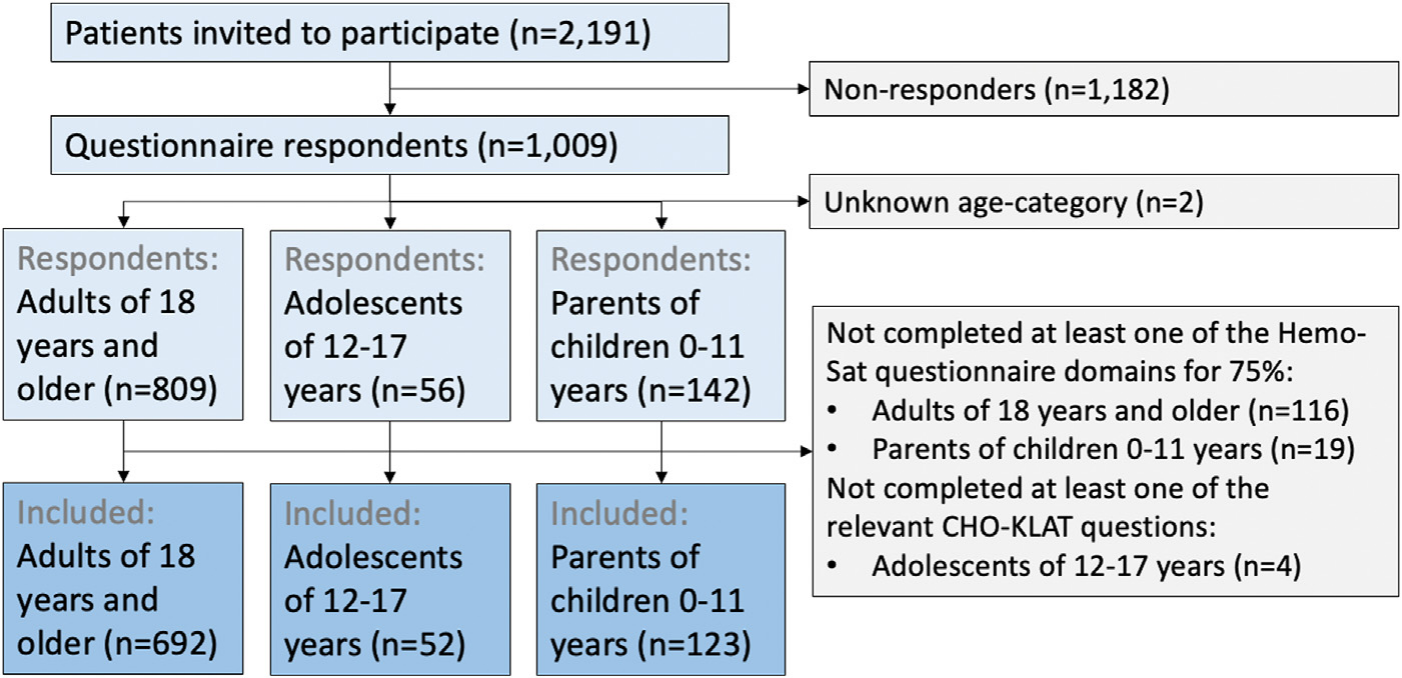
Study flowchart of the 867 included participants. CHO-KLAT, Canadian Hemophilia Outcomes Kids’ Life Assessment Tool.

The HiN6 questionnaire covered several aspects of hemophilia. For the current study, a subset of data on hemophilia care satisfaction was used. Respondents who completed relevant questions on care satisfaction were included, as illustrated by the study flowchart (Figure 1).

#### 2.1.3 Measures - Patient satisfaction with hemophilia care (Hemo-Sat, CHO-KLAT, and VAS scales)

To assess satisfaction among adults and parents of children aged 0 to 11 years, the validated Hemophilia Patient Satisfaction Scale (Hemo-Sat) questionnaire was used [14,15]. Hemo-Sat is a hemophilia-specific care satisfaction questionnaire for adults (version Hemo-SatA, 34 items) and parents of young children (version Hemo-SatP, 35 items). Hemo-Sat has been validated in 28 languages [16]. Questions are divided into 6 domains: ease and convenience, efficacy, burden, specialist/nurse, center/hospital, and general satisfaction. Answers are given on a 5-point Likert scale, ranging from total agreement to total disagreement on statements. Both total and domain scores were analyzed, as well as individual item responses. Each standardized (domain) score ranged from 0 to 100, with lower scores indicating higher satisfaction. For each Hemo-Sat domain, a minimum data completion rule of 75% was applied. Participants who completed at least 1 Hemo-Sat domain were included. Hemo-Sat’s reliability is high (Cronbach’s α = 0.71-0.95, assessed among adults) [15,17].

Because Hemo-Sat was not validated for adolescents aged 12 to 17 years, 5 items from the Canadian Hemophilia Outcomes Kids’ Life Assessment Tool (CHO-KLAT) were used for the assessment of care satisfaction [18]. CHO-KLAT is a hemophilia-specific questionnaire for children aged 4 to 18 years to evaluate health-related quality of life in the previous 4 weeks. Of 35 items, 5 evaluate care satisfaction. All adolescents who completed at least 1 of these 5 items were included. Answers were given on a 5-point Likert scale, ranging from never to always. CHO-KLAT’s reliability is high among boys aged 8 to 16 years (Cronbach’s α = 0.81-0.91) [19,20].

Adults and parents of children aged 0 to 11 years were asked the following question: “How would you rate your care in its totality for the past 12 months?” Grades were given on a visual analog scale (VAS) from 1 (lowest) to 10 (highest).

#### 2.1.4 Measures - Factors determining care satisfaction (open questions)

First, in 2 open questions, adults and parents were asked to comment on their satisfaction score on the VAS scale. Second, they were asked the following question: “Is there anything else you would like to say about your care?”

#### 2.1.5 Measures - Healthcare improvements (multiple choice and open questions)

We included 1 multiple choice question with 8 answer options: “If you were able to improve one area of your care, which would it be?” Participants could choose between the following options: information provision, shared decision making, coordination between health care providers, accessibility of institutions, discussing all relevant topics, respectful patient-professional relationships, health care costs, and personal relationships with health care providers.

In one open question, adults and parents commented on their answer in the multiple choice question.

#### 2.1.6 Data analysis

Descriptive statistics were used and reported as mean values with 95% CI or median values with IQR. Education level was used as a sociocultural determinant of health [21]. To evaluate the effects of patient characteristics on satisfaction, Mann–Whitney U-tests were used. *P* values of <.05 were considered significant. Answers on open questions were analyzed using the software program MAXQDA by MB, using thematic content analysis.

#### 2.1.7 Ethics

Approval was obtained from the Institutional Review Board of the Leiden University Medical Center (NL59114.058.17). Participants who completed the questionnaire were considered to consent.

### 2.2 Qualitative study

Through semistructured interviews set between September 2020 and February 2021, participants were asked about their perceptions on factors determining care satisfaction and potential health care improvements.

#### 2.2.1 Participants

Study participants were Dutch adult and pediatric patients with hemophilia A or B and their parents and multidisciplinary health care providers working within and outside of comprehensive hemophilia care centers. To include otherwise underrepresented female perspectives due to the X-linked genetic inheritance of hemophilia, we interviewed women with other inherited coagulation disorders. Theoretical sampling was used to include a diverse set of participants.

Patients and parents were recruited in 2 CCCs, the Amsterdam University Medical Center and Erasmus Medical Center, and through open invitations spread by the Dutch Hemophilia Patient Society using their website, email newsletter, and Facebook page. Health care providers were recruited from CCCs in the Netherlands. We continued participant inclusion until thematic saturation was achieved; no new information was introduced in the last 2 interviews.

#### 2.2.2 Data collection

Interviews were conducted by 2 male junior clinician-scientists: M.B., a physician-researcher, and J.M., a physiotherapist-researcher. A part of the interviews was conducted in person and the remaining part using video conferencing due to COVID-19 restrictions. Duration of interviews was 44 to 96 minutes. All interviews were audiotaped and transcribed verbatim. Interviews were conducted using an item topic list based on HiN6 questionnaire study results and literature. After discussing relevant personal details, participants were asked about their perspectives on hemophilia care and what elements determine this, including the communication between health care providers. Next, their perspectives on health care improvements were discussed, including perspectives on digital health tools and teleconsulting.

#### 2.2.3 Data analysis

All interviews were analyzed using MAXQDA. Themes were assessed using a directed form of thematic content analysis because elements of our coding scheme were predetermined by our research question. M.B. coded all interviews and J.M. coded a third of the interviews. Both drafted the coding scheme. Final thematic discussions were done by M.B. and J.M. with senior clinician-scientists L.H. and S.G.

#### 2.2.4 Ethics

Approval was given by the Institutional Review Board of the Amsterdam University Medical Center (W20_383 # 20.428). All participants signed an informed consent form.

## 3 Results

### 3.1 Quantitative study

#### 3.1.1 Participant characteristics

The overall questionnaire response rate was 46% (1009 of 2191), as illustrated in the study flowchart (Figure 1). Respondents were similar to the overall Dutch hemophilia population in terms of age and disease severity [4]. Respondents’ education level as well as their socioeconomic participation were similar to those of the overall Dutch population [22,23]. Of respondents, 80% were adults, 6% were adolescents, and 14% were parents of children aged 0 to 11 years. Relevant data for the current study were available for 867 participants. Participant characteristics are presented in Table 1. Included participants were similar to overall HiN6 respondents in terms of the proportion with severe hemophilia (39% vs 38%), median age (44 vs 40 years), and prophylaxis use (38% vs 36%) [4]. Of study participants with severe hemophilia, 91% used prophylaxis. Of children under the age of 18 years with severe hemophilia, 97% used prophylaxis. Since nonfactor replacement therapy was not readily available at the time of this study, most patients used factor VIII or factor IX prophylaxis.

**Table 1:** Characteristics of included questionnaire participants.

Characteristics	All included participants (n = 867)	Adults (n = 692)	Adolescents aged 12-17 y (n = 52)	Parents of children aged 0-11 y (n = 123)
Age (y), median (IQR)	44 (22-60)^a^	50 (33-62)^b^	14 (13-16)	6 (3-9)^b^
Hemophilia A, n (%)	754 (87%)^b^	605 (87%)^c^	43 (83%)^d^	106 (86%)
Severe hemophilia, n (%)	335 (39%)	246 (36%)	32 (62%)	57 (46%)
Prophylaxis, n (%)	331 (38%)^b^	237 (34%)^c^	31 (60%)^d^	62 (50%)
No. of HCP,^e^ median (IQR)	4 (3-6)	4 (3-6)	4 (3-5)	3 (2-4)
HIV infection, n (%)				
Current infection	22 (3%)	22 (3%)	0 (0%)	0 (0%)
Hepatitis C infection, n (%)				
Current infection	8 (1%)	8 (1%)	0 (0%)	0 (0%)
Past infection	223 (26%)	223 (32%)	0 (0%)	0 (0%)
Inhibitory antibodies, n (%)				
Current inhibitor	14 (2%)	12 (2%)	0 (0%)	2 (2%)
Past inhibitor	88 (10%)	63 (9%)	5 (10%)	20 (16%)
Education level,^f^ n (%)				NA
Lower	189 (25%)	168 (24%)	21 (40%)	
Intermediate	237 (32%)	220 (32%)	17 (33%)	
Higher	291 (39%)	277 (40%)	14 (27%)	
Unknown	27 (4%)	27 (4%)	0	

Data on ethnicity were not collected because it is not allowed under Dutch law.

HCP, health care provider; NA, not applicable.

a Six missing variables.

b Three missing variables.

c Two missing variables.

d One missing variable.

e The self-reported number of health care providers that participants had been in contact with during the 12 months prior to filling out the questionnaire.

f For adults, the highest completed education level is reported. For adolescents, the highest current education level is reported. For adults, the lower education level includes primary education, prevocational secondary education (VMBO), lower secondary vocational training (MBO-1), and the first 3 years of senior general secondary education (HAVO) and preuniversity secondary education (VWO). The intermediate education level includes upper secondary education (HAVO/VWO) and vocational and middle management training (MBO-2, MBO-3, and MBO-4). Higher education includes Bachelor and Master degree programs at universities of applied sciences (HBO), research universities (WO), and doctoral degree programs. For adolescents, the lower education level includes prevocational secondary education (VMBO) and secondary vocational training (MBO). The intermediate education level includes senior general secondary education (HAVO) and vocational and middle management training (MBO). The higher education level includes preuniversity secondary education (VWO).

#### 3.1.2 Patient satisfaction with hemophilia care

**Figure 2:**
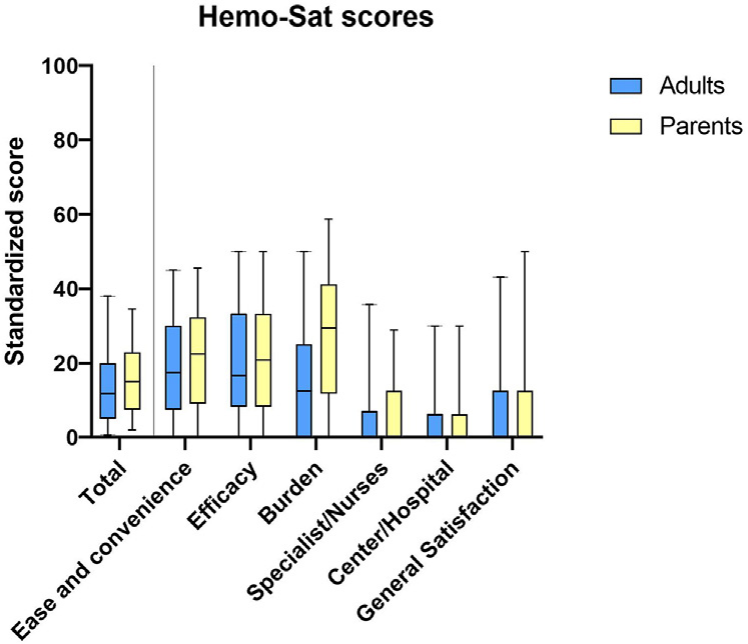
Standardized Hemo-Sat questionnaire domain scores for each of the 6 Hemo-Sat domains and the total score. Boxplots indicate median (IQR) values; whiskers indicate the 95% CI. Lower scores indicate higher satisfaction.

The median (IQR) overall Hemo-Sat score of 704 adults and parents of children aged 0 to 11 years was 12 (6-21). The results are shown in Figure 2. Values are presented in Supplementary Table 1. The highest satisfaction was expressed on the 3 Hemo-Sat domains: “general satisfaction,” “specialist/nurse,” and “center/hospital.” Overall, 96% (737 of 764) of adults and parents reported that they were satisfied or very satisfied with their care. Lower satisfaction was expressed on the domains “efficacy,” “burden,” and “ease and convenience.” Of adults and parents, 63% (446 of 711) reported that they were (very) confident that adequate prophylaxis administration could prevent bleeds and 12% (99 of 797) reported that treatment (strongly) interfered with everyday life. On the domain “burden,” lower satisfaction was expressed by parents compared to adults, with respective Hemo-Sat scores of 30 (12-41) and 13 (0-25). This difference could be attributed to one question: how much participants worried about receiving injections. Twelve percent (73 of 630) of adults expressed (strong) worries), opposed to 40% (46 of 115) of parents.

Differences in Hemo-Sat scores were observed between subgroups. For the domain “ease and convenience,” differences were observed between participants who were treated with prophylaxis and on-demand therapy and between participants with severe and nonsevere hemophilia. For the domain “burden,” these differences were only observed among parents of children. Data are presented in Supplementary Table 2.

Of adolescents, 87% (27 of 31) were often or always satisfied with their treatment. Coagulation factor infusions were often or always considered burdensome by 8% (2 of 26). Other treatment aspects, including resting after a bleed, were often or always considered burdensome by 19% (4 of 21).

Adults rated their care with a mean VAS score of 8 (95% CI, 6-10; n = 573). For 99 parents of young children aged 0 to 11 years, this was 8 (95% CI, 7-10).

#### 3.1.3 Factors determining care satisfaction

In total, 449 participants responded to open questions. Many patients described their long-lasting bonds with health care providers (Table 2, quotes Q1 and Q2). Seventy-nine adults argued that health care costs were too high. The majority (90%, 71 of 79) referred to the yearly recurrent individual deductible fee, which they considered unjustly high for chronically ill patients (Q3). A minority (10%) referred to the financial impact on society (Q4). Additionally, 42 participants expressed that collaborations between professionals working within and outside of a CCC are sometimes suboptimal (Q5 and Q6). Fifteen participants signaled a lack of knowledge on hemophilia among professionals not working within CCCs (non-CCC professionals) (Q7).

**Table 2:** Questionnaire quotes on elements determining hemophilia care satisfaction.

Theme	No.	Quote
Patient-professional interactions	Q1	“Healthcare providers are extraordinarily engaged, professional and friendly.”—Male, aged 30-49 y, with severe hemophilia B
	Q2	“A healthcare team which is easily approachable, thoughtful and caring. Truly great.”—Parent of a son, aged 6-12 y, with severe hemophilia A
Health care costs	Q3	“Paying deductible health insurance costs as a chronically ill person is ridiculous.”—Male, aged 18-29 y, mild hemophilia A
	Q4	[Desire] “Reduction in medication prices, which are insanely high, so I feel less guilt towards society when using it.”—Male, aged 50-65 y, with severe hemophilia A
Collaborations between health care providers	Q5	“Care is good. However, collaborations and coordination between hospitals and primary care, and between healthcare providers in different institutions could be better.”—Male, aged 30-49 y, with severe hemophilia A
	Q6	“When you are treated in multiple healthcare institutions and your symptoms are not straight-forward, it would be nice if healthcare providers would consult one another, instead of expecting a patient to act as some sort of intermediary.”—Male, aged 30-49 y, with severe hemophilia A
	Q7	“The Hemophilia Treatment Center is great. However, the emergency department delivers poor care. Me and my son had to wait for five hours before he received his coagulation medication. Even though I repeatedly expressed my worries and mentioned he has hemophilia!”—Parent of a son, aged 6-12 y, with mild hemophilia A
Information provision	Q8	[Desire] “More information about new treatment options, such as gene therapy.”—Parent of a son, aged 0-5 y, with mild hemophilia A

#### 3.1.4 Health care improvements

**Figure 3:**
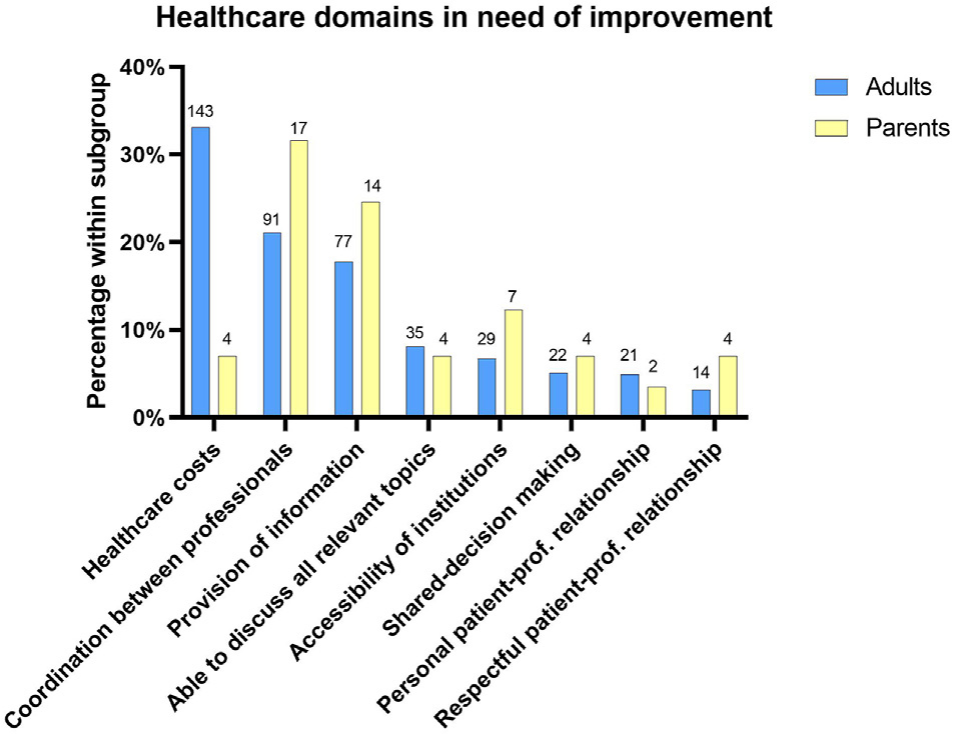
Participant’s responses on the multiple choice question “If you could improve one area of your care, which would it be?” Absolute numbers are presented above bars. Percentages indicate how many participants chose an answer option as a fraction of all participants that answered this question.

If questionnaire participants could improve 1 area of their care, 33% (143 of 432) of adults would lower health care costs (Figure 3). Both adults and parents would also enhance coordination between health care providers (91 of 432 [21%] and 17 of 56 [30%], respectively) and improve information provision (77 of 432 [18%] and 14 of 56 [25%], respectively).

In describing care improvements, 53 adults and parents expressed a desire to be more frequently and extensively informed on novel treatment options (Q8). Several patients suggested that digital health tools could assist with this. Some patients suggested that improvements in health information exchange would aid the sometimes lacking communication between professionals working within and outside CCCs. Finally, teleconsulting was suggested to further increase the approachability of care institutions.

### 3.2 Qualitative study

#### 3.2.1 Participant characteristics

Of 19 patients, 14 (74%) were male. Their median (IQR) age was 39 (18-65) years, 58% had severe hemophilia, and 63% used prophylaxis. One participant had von Willebrand disease type 1, and 1 had factor VII deficiency. Five participants were parents of a child with hemophilia. The 18 participating health care providers reflected all members of comprehensive hemophilia care teams. Sixteen (89%) were female, and the median (IQR) work experience was 16 (8-23) years. Characteristics are presented in Table 3.

**Table 3:** Characteristics of interview participants.

Characteristics	Patients and parents (n = 19)	Health care providers (n = 18)
Sex, n (%)	Male, 14 (74%)	Male, 2 (11%)
Age (y), median (IQR)^a^	39 (18-65)	NA
Work experience (y), median (IQR)	NA	16 (8-23)
Condition, n (%)	Mild and moderate hemophilia, 6 (32%)	NA
	Severe hemophilia, 11 (58%)	
	von Willebrand disease, 1 (5%)	
	Factor VII deficiency, 1 (5%)	
Use of prophylaxis, n (%)	Yes, 12 (63%)	NA
Comorbidities, n (%)	Many, 6 (32%)	NA
	Some, 2 (11%)	
	None, 11 (58%)	
Role or function, n (%)^b^	Patient, 14 (74%)	(Pediatric) hematologist, 5 (28%)
	Caretaker, 5 (26%)	(Specialized) hemophilia nurse, 5 (28%)
	With child, 2 (10%)	Infectiologist, 1 (6%)
	Without child, 3 (16%)	Orthopedist, 1 (6%)
		Physiotherapist, 2 (11%)
		Psychologist, 1 (6%)
		Social worker, 1 (6%)
		General practitioner, 1 (6%)
		Pharmacist, 1 (6%)
Digital expertise, n (%)	Proficient, 9 (47%)	Proficient, 7 (39%)
	Average, 6 (32%)	Average, 6 (33%)
	Not proficient, 4 (21%)	Not proficient, 5 (28%)
Used teleconsulting, n (%)	Yes, 5 (26%)	Yes, 10 (56%)
Accessed patient portal, n (%)	Yes, 7 (37%)	NA

NA, not applicable.

a Ages represent those of children, adolescents, and adults, not of adults who filled in the questionnaire.

b Some participants were both patient and caretaker. Their most prominent role, as discussed in the interview, is stated here.

#### 3.2.2 Factors determining care satisfaction

In discussing which factors determine participant’s care satisfaction, 3 overarching themes were identified from patient and professional interviews: (I) patient-professional interactions, (II) availability of care, and (III) coordination of care. Interview quotes are shown in Table 4. Without exception, participants made a strong distinction between care initiated within CCCs (CCC care) and outside of it (non-CCC care), as explained in Box B.

**Table 4:** Interview quotes on hemophilia care satisfaction.

Theme	Within comprehensive care centers	Outside comprehensive care centers
(1) Patient-professional interactions	Q9 “A hemophilia department: it still works perfectly. A small group of people. They all know me by my full name.”—Male patient, aged >65 y, with mild hemophilia A.	Q11 “Well, often I have to convince healthcare providers to do it [contact a hemophilia treatment center for collaboration]. Which is not that simple. Especially when dealing with experts.”—Male patient, aged >65 y, with severe hemophilia A.
	Q10 “Part of strong communication originates from healthcare providers that know each other. Colleagues working within a single hospital know one another.”—Hematologist with >20 y of experience.	
(2) Availability of care	Q12 “They are very accessible and easy to call. […] I never feel like I’m bothering them. I can always contact them; they’ve stressed that repeatedly.”—Parent of son, aged 6-12 y, with severe hemophilia A.	Q14 “So, I was taken to the emergency department. But I had to talk and talk and talk in order to be taken to the hemophilia treatment center. They wanted to bring me to a local hospital! […] Only after they talked to a higher-ranking physician, they agreed to bring me there.”—Male patient, aged 50-65 y, with moderate-to-severe hemophilia A.
	Q13 [On the need for psychosocial support] “This sounds very brave: me taking control. But actually, as a patient, it’s often quite difficult… Especially when you’ve already established a strong relationship with your doctor, to indicate that care is lacking.”—Female patient, aged 30-49 y, with inherited bleeding disorder.	
(3) Coordination of care	Q15 “All these different parties communicate really well. The physiotherapist contacts the doctor, who contacts the general practitioner. There is a lot of communication going on between them. And it’s going smoothly.”—Parent of a son, aged 6-12 y, with severe hemophilia A.	Q16 “Miscommunications between healthcare providers are common when patients are treated in different institutions. And it happens. Patients with mild hemophilia that have little control over their disease. They can be in contact with many providers in many institutions. And yes, that is very difficult.”—Hematologist with 11-20 y of experience.
		Q17 “The first thing I tell them [healthcare providers] is: ‘Pay attention!’ Because I never expect a healthcare provider to know I have hemophilia.”—Male patient, aged 50-65 y, with moderate-severe hemophilia A.
		Q18 “Pharmacies don’t have my medication in stock… Nurses don’t know exactly how to administer all types of medication… It all makes sense. But still, as a patient, it not always makes you feel safe.”—Female patient, aged 30-50 y, with inherited bleeding disorder.
		Q19 “Of course, the problem is that no one is ultimately responsible for a patient in contact with multiple doctors.”—Male patient, aged >65 y, with severe hemophilia A.
		Q20 “Everybody knows that currently, the exchange of medical information is a mess… is in need of improvement. […] This is the biggest bottleneck when talking about the quality of care at this moment.”—Hematologist with 0-10 y of experience.

Box B **Comprehensive care centers care vs non–comprehensive care centers care.**

Care initiated within comprehensive care centers (CCCs) (CCC care) refers to all specialized and multidisciplinary care delivered within CCCs and care routes initiated by CCC professionals, including multidisciplinary consultations with non-CCC professionals. Non-CCC care refers to all care delivered outside of CCCs, including primary care and hospitals without a CCC, and care routes initiated by non-CCC professionals. Therefore, we elaborated on the 3 themes separately for CCC care and non-CCC care.

Regarding patient-professional interactions, all patients were very satisfied with their health care providers working in CCCs and described strong patient-professional bonds (Q9). Multiple professionals added that the teamwork within CCCs is what defines hemophilia care and solidifies the quality of care (Q10). In contrast, multiple patients said that they did not always feel taken seriously by professionals inexperienced in treating hemophilia (Q11).

Regarding availability of care, 4 subthemes were identified. First, most patients indicated that the availability of care within CCCs is excellent (Q12). Only a small minority had difficulties reaching their CCC by phone outside of office hours. Second, patients considered the support of allied professionals to be readily available within their CCCs, including physiotherapists and psychologists. A few adults disputed this and signaled that long-lasting patient-professional bonds made it more difficult to declare a desire for psychosocial support (Q13). These patients would advise professionals in adult care to offer psychosocial support more actively. Third, some patients said that travel distances from home to the nearest CCC are quite large. They related this to the centralization of hemophilia care. Finally, several professionals expressed uncertainty over the future availability of care, considering rising costs due to new treatment options.

Regarding non-CCC care, several patients reported that they occasionally had to plea for CCC referral in case of emergency, when contacting non-CCC professionals inexperienced in treating hemophilia (Q14).

Regarding coordination of care, nearly all patients said that the collaboration of all professionals working within CCCs was excellent. The concentration of expertise and the coordinating role of (specialized) hemophilia nurses were considered valuable. Especially for patients with few comorbidities, this was said to result in well-coordinated care (Q15). Professionals agreed with patients’ views. However, most patients said that communication between professionals working within and outside of CCCs is often limited. This especially had a negative impact on patients with many care providers (Q16).

Four subthemes regarding non-CCC coordination of care were identified. First, a large majority of adult patients indicated that they never expect a non-CCC professional to be aware of their medical history (Q17). Both patients and professionals signaled a lack of hemophilia knowledge among non-CCC professionals. Second, most patients with comorbidities said that they must take control in order to prevent harmful consequences of actions initiated by non-CCC professionals, such as treatment initiation or (lack of) referrals. Although many felt accustomed to this, some admitted that this occasionally made them feel unsafe (Q18). Both patients and professionals recognized that not every patient is able to coordinate care, especially patients with lower health literacy or those who find it difficult to make themselves heard. It was said that patient participation in coordinating care should remain optional. Third, a few patients said that not all professionals are open toward patients taking control. Two solutions for coordination issues were suggested: to better define responsibilities of patients and professionals, and to formally appoint a professional who is ultimately responsible for coordination (Q19). Professionals added the need to urgently solve the technical limitations in the exchange of medical information between health care providers (Q20). Finally, female patients and their health care providers signaled that female bleeding symptoms are more often underrated than male symptoms, resulting in a diagnostic delay. For example, heavy menstrual bleeding is sometimes considered a family trait. These participants suggested that improved interprofessional collaborations would aid early signaling of symptoms.

#### 3.2.3 Health care improvements

While discussing care satisfaction, patients and professionals suggested multiple digital solutions to improve care: improved digital exchange of medical data, teleconsulting to improve approachability, and digital tools to aid information provision. Patients listed all tools they currently use: teleconsulting tools, patient portals, and a digital treatment diary to log medication use and bleeding episodes (VastePrik; HemoNED). Pediatric patients often used a questionnaire portal to complete patient-reported outcomes measures (KLIK; Department of Child and Adolescent Psychiatry & Psychosocial Care, Emma Children's Hospital) [24]. In evaluating current and potential future digital health tools, 4 themes were identified: (I) increased insight, (II) inclusiveness of care, (III) complementary to usual care, and (IV) technical and usability prerequisites. Themes are explained in Box C, and interview quotes are shown in Table 5. Both patients and professionals generally considered the increased use of digital health tools to be of added value and a logical element in health care innovation.

Box C **Themes regarding current and potential future digital health tools.**

I. Increased insight: A majority of patients said that digital tools and patient portals increased their understanding of hemophilia and its impact on life (Q21). Both patients and professionals mentioned that these tools help focus and improve discussions in check-up visits. Several patients reported that it helped them to articulate otherwise ignored symptoms (Q22).
II. Inclusiveness of care: Nearly all participants emphasized the importance of safeguarding the inclusiveness of care. They referred to patients who are less digitally proficient, who have trouble managing their care, who are dismissive of technology, or for whom the use of digital tools is not feasible, such as young children (Q23/Q24). People should always be able to ask for support with the use and interpretation of digital tools. Currently, many patients have already experienced difficulties in interpreting patient portal information (Q25). They expressed their concerns regarding their participation in our increasingly complex and digital-oriented society.
III. Complementary to usual care: A vast majority of participants warned that digital health tools and teleconsulting should not replace face-to-face care, but be complementary (Q26). Even though nearly all patients acknowledged its flexibility and reduction in travel times, no one wanted to fully replace traditional contacts. Several patients suggested that digital tools should be demand-driven: available when needed and possible to disregard if not. A risk of telemonitoring was formulated by some professionals: too much monitoring could lead to the undermining of a patient’s trust and autonomy. Finally, to minimize information loss, a few professionals formulated a “minimal functioning level” of a patient-professional relationship before patients could be considered ready to use teleconsulting (Q27).
IV. Technical and usability prerequisites: Nearly all participants expected digital health tools to be of the highest quality regarding user friendliness, technical design, helpdesk support, and interoperability with other tools (Q28). Many participants said that even minor problems would result in dropouts, as is the case with current digital health tools.

**Table 5:** Interview quotes on remote health care tools.

Theme	No.	Quote
Increased insight	Q21	[Evaluating the digital treatment diary] “It really helped me understand, especially the graphs. To verify: in a year, how many bleeding episodes did I have? And of what type? It really opened my eyes. I am always inclined to say: ‘Well, it is going okay’. But when I’d sum up all bleeding episodes, I’d realize I’ve had ten in a year!”—Female patient, aged 30-50 y, with inherited bleeding disorder.
	Q22	[On teleconsulting] “It can even be quite useful, because… because of this barrier which is present. That can actually be very helpful. Especially for men, so they can open up a bit more easily.”—Social worker with 11-20 y of working experience.
Inclusiveness of care	Q23	“For regular outpatient clinic appointments, I believe video conferencing is inferior to seeing patients in person. You receive less information. Especially for those patients where, I believe, there is a discrepancy in the perception of whether things are going well. And by that, naturally, I refer to our adolescent patients.”—Hematologist with >20 y of experience.
	Q24	“The danger in these kinds of applications… the biggest danger is that we are only improving care for those that already have good access to it. The more e-Health tools we develop, and the fancier they become, the more this may become apparent.”—Psychologist with 0-10 y of experience.
	Q25	[On laboratory results in a patient portal] “Those results. I think to myself: what do they mean? I have no idea.”—Male patient, aged >65 y, with severe hemophilia A.
Complementary to usual care	Q26	“I’ve been injecting a lot of medication lately. But why? Because of my illness? Or because I went boxing once? An app won’t show you this. Data requires context. And this context becomes apparent through conversation.”—Male patient, aged 30-50 y, with severe hemophilia A.
	Q27	“You’d actually want to have a scoring system that characterizes a relationship. The value, which defines the quality of the patient-professional relationship… that value should be higher than a certain threshold level, before you can switch to video consultations. […] And one patient matched with one professional reaches this threshold faster than another set.”—Pediatric hematologist with >20 y of experience.
Technical and usability preconditions	Q28	“Image I need six screens when talking to a patient? I wouldn’t even be able to see my patients; they would sit behind those screens! When I would need to check all of those [apps and tools], I wouldn’t even be looking at my patient anymore. We should be cautious… we should aim to integrate things.”—Hematologist with >20 y of experience.

## 4 Discussion

### 4.1 Patient satisfaction with hemophilia care

This study quantitatively and qualitatively evaluated perspectives of the Dutch hemophilia population on the quality of their care. We found that overall care satisfaction was high, similar to that in several international studies. In a US questionnaire study, between 94% and 98% of persons with hemophilia reported to be satisfied with CCC care [25]. In a German questionnaire study, 96% of participants were either satisfied or very satisfied with their care [26].

In the questionnaire study, 2 health care domains were evaluated most positively by patients: “specialist/nurse” and “center/hospital.” These positive aspects related to patient-professional communication, treatment explanations, and availability of care. The domains “efficacy,” “burden,” and “ease and convenience” were ranked less favorably, primarily due to the high burden associated with repeated intravenous administration of therapy. This finding is supported by previous research. In a Dutch qualitative study, interviewed patients said that self-administering treatment can be challenging [27]. In a mixed methods study among German persons with hemophilia and their parents, 34% would switch to a new product for easier administration [28]. However, since nonfactor replacement therapy was not readily available at the time of this study, this identified burden is expected to improve [4].

To date, no studies have compared satisfaction among adults and parents. We found that children worry more about receiving injections compared to adults. We suggest 2 hypotheses. First, it is known that fear of needles is more prevalent among young children [29]. Second, older participants might consider current treatment administration easier in comparison to the past and thus express higher satisfaction [27].

Finally, the observed differences in Hemo-Sat domain scores between subgroups with different treatment regimens and hemophilia severity suggest that different patients experience the organization of their care differently and have different needs.

### 4.2 Factors determining care satisfaction

Factors determining patient satisfaction that were identified in open questions matched those identified in interviews and were related to patient-professional interactions, availability of care, and coordination of care. In both the questionnaire and interviews, a clear distinction was made between care initiated or delivered within and outside of CCCs. Many patients and professionals related this difference to the centralization of hemophilia care. While centralization resulted in well-organized CCCs, it also led to a lack of knowledge among other professionals. This observation was supported by a qualitative study among persons with hemophilia in the United States, who reported limited knowledge among non-CCC professionals, including emergency room staff [30].

Albeit a logical and perhaps inevitable consequence, this creates a remarkable paradox. To compensate for the lack of knowledge among non-CCC professionals, patients are expected to be alert and take on a coordinating role, especially those in contact with many different health care providers. Patients often expressed resistance toward this role but also indicated a certain habituation in doing so.

### 4.3 Health care improvements

Two potential health care improvements were suggested by participants. Stressing these additional care improvements is especially relevant in the current era of treatment innovation, in which other aspects of care might be less focused upon. First, the use of digital tools for more frequent and in-depth information provision was often suggested. Second, regarding care coordination, most patients did not necessarily disregard their role as active conductors of their care. Instead, they suggested 4 conditions to make coordination more manageable. First, coordination should be optional since not all patients can and/or want to carry out this role. Second, more supporting tools were considered necessary to facilitate self-management and approachability, including telemonitoring and teleconsulting. Third, participants suggested to formally appoint a health care provider who is ultimately responsible for coordination and to better define patient and professional responsibilities. Finally, improved health information exchange between different professionals was considered essential to facilitate coordination. This was corroborated by a Dutch questionnaire study among medical specialists [31] and a systematic review evaluating health information exchange in the United States [32].

Finally, in evaluating digital health tools, patients and professionals expressed heterogeneous views. This illustrates the plurality of perceptions on this newly emerging topic. Conflicting views became apparent between digitally proficient and less proficient users and between participants with high and low health literacy. This underlines the need for care to remain inclusive. As some health care providers stated, “we have a responsibility to preserve accessibility.” Health care providers should make sure to not merely improve care for those who already have good access to care and risk leaving behind those who do not.

### 4.4 Strengths and limitations

A strength of this study is the combination of quantitative and qualitative methods. This enabled us to focus interview questions on the topics identified from the questionnaire. The first limitation of the questionnaire study is the potential selection of patients who completed at least 1 Hemo-Sat domain. This may have led to an overestimation of treatment satisfaction because these patients might be more involved in their care than the general population. We were unable to report race or ethnicity as a sociocultural determinant of health but did report education level. Second, Hemo-Sat has not been validated for children aged between 12 and 17 years, resulting in a partial evaluation of pediatric care. Third, in general, patients tend to report high satisfaction with their health care providers, which partly impedes critical judgments [33]. Yet, by combining different study methods and focusing on care improvements, we aimed to address this. Finally, there might be considerable interindividual heterogeneity in reported satisfaction. We addressed this by including a large number of patients to obtain reliable estimates, using validated questionnaires, and combining quantitative and qualitative data collection.

A limitation of the interview study might be that follow-up questions on interprofessional communication and digital tools could have directed participants’ answers. However, since interview topics were based on questionnaire responses and most interviewees mentioned these topics spontaneously, we do not believe that this greatly affected the results. Second, participants volunteering in interviews could represent a subgroup with increased interest in digitalization or research. Third, the survey was conducted before the COVID-19 pandemic, as opposed to midpandemic interviews, which could have altered perspectives, especially on digital solutions. Finally, at the time of the questionnaire, only 9.4% of patients had switched to extended half-life products and 0.8% used nonfactor replacement therapy [4]. During interviews, more participants started using these treatments, which could have influenced perspectives. Future increases in the use of nonfactor replacement therapy will likely further lower the experienced treatment burden.

## 5 Conclusion

Overall, people with hemophilia in the Netherlands are very satisfied with their care. Still, patients and health care providers suggested to improve the coordination of care, especially for patients in contact with many different health care providers or with providers inexperienced in treating people with hemophilia. Both patients and professionals anticipated that digital health tools might help in achieving this.

## Appendix

The SYMPHONY consortium is coordinated by Marjon H. Cnossen (Rotterdam, the Netherlands) and Simone H. Reitsma (Rotterdam, the Netherlands). The HiN6 Steering Group includes Lize F. D. van Vulpen (Nijmegen, the Netherlands), Jeroen Eikenboom (Leiden, the Netherlands), Erik A. M. Beckers (Maastricht, the Netherlands), Louise Hooimeijer (Groningen, the Netherlands), Paula F. Ypma (the Hague, the Netherlands), Laurens Nieuwenhuizen (Eindhoven, the Netherlands), Michiel Coppens (Amsterdam, the Netherlands), Saskia E. M. Schols (Nijmegen, the Netherlands), Britta A. P. Laros (Nijmegen, the Netherlands), Paul R. van der Valk (Utrecht, the Netherlands), Marjon H. Cnossen (Rotterdam, the Netherlands), Mariëtte H. E. Driessens (Nijkerk, the Netherlands), Johanna G. van der Bom (Leiden, the Netherlands), Frits R. Rosendaal (Leiden, the Netherlands), Cees Smit (Leiden, the Netherlands), Frank W. G. Leebeek (Rotterdam, the Netherlands), and Samantha C. Gouw (Amsterdam, the Netherlands). Shermarke Hassan (Leiden, the Netherlands) and Erna C. van Balen (Leiden, the Netherlands) helped develop the HiN6 protocol and collected and analyzed the data. Johanna G. van der Bom, Cees Smit, Frits R. Rosendaal, and Samantha C. Gouw initiated and coordinated the HiN6 project and supervised data collection.

## References

[1] Plug I., Van der Bom J.G., Peters M., Mauser-Bunschoten E.P., De Goede-Bolder A., Heijnen L. (2004). Thirty years of hemophilia treatment in the Netherlands, 1972-2001. Blood.

[2] Mannucci P.M. (2020). Hemophilia therapy: the future has begun. Haematologica.

[3] Hassan S., Monahan R.C., Mauser-Bunschoten E.P., van Vulpen L.F.D., Eikenboom J., Beckers E.A.M. (2021). Mortality, life expectancy, and causes of death of persons with hemophilia in the Netherlands 2001-2018. J Thromb Haemost.

[4] Hassan S., van Balen E.C., Smit C., Mauser-Bunschoten E.P., van Vulpen L.F.D., Eikenboom J. (2021). Health and treatment outcomes of patients with hemophilia in the Netherlands, 1972-2019. J Thromb Haemost.

[5] van Balen E.C., Krawczyk M., Gue D., Jackson S., Gouw S.C., van der Bom J.G. (2019). Patient-centred care in haemophilia: patient perspectives on visualization and participation in decision-making. Haemophilia.

[6] Big Data Institute, University of Oxford Patient reported outcome measures. http://phi.uhce.ox.ac.uk/inst_types.php.

[7] Hoefnagels J.W., Schrijvers L.H., Leebeek F.W.G., Eikenboom J., Schols S.E.M., Smit C. (2021). Adherence to prophylaxis and its association with activation of self-management and treatment satisfaction. Haemophilia.

[8] Butler R.B., Cheadle A., Aschman D.J., Riske B., Senter S., Mclaughlin K.M. (2016). National needs assessment of patients treated at the United States Federally-Funded Hemophilia Treatment Centers. Haemophilia.

[9] Batbaatar E., Dorjdagva J., Luvsannyam A., Savino M.M., Amenta P. (2017). Determinants of patient satisfaction: a systematic review. Perspect Public Health.

[10] Gavurova B., Dvorsky J., Popesko B. (2021). Patient satisfaction determinants of inpatient healthcare. Int J Environ Res Public Health.

[11] McDonald K., Sundaram V., Bravata D.M. (2007).

[12] Watson R. (2012). Netherlands tops European healthcare league, with UK coming in at 12th. BMJ.

[13] The Commonwealth Fund. Mirror, mirror 2021: reflecting poorly. Health care in the U.S. compared to other high-income countries. https://www.commonwealthfund.org/publications/fund-reports/2021/aug/mirror-mirror-2021-reflecting-poorly.

[14] Gringeri A., Von Mackensen S. (2008). Quality of life in haemophilia. Haemophilia.

[15] Gringeri A., Mantovani L., Mackensen S.V. (2006). Quality of life assessment in clinical practice in haemophilia treatment. Haemophilia.

[16] von Mackensen S., Campos I.G., Acquadro C., Strandberg-Larsen M. (2013). Cross-cultural adaptation and linguistic validation of age-group-specific haemophilia patient-reported outcome (PRO) instruments for patients and parents. Haemophilia.

[17] von Mackensen S., Gringeri A., Scalone L., Mantovani L. (2005). Group the CS. How satisfied are patients in Italy with the treatment of their haemophilia? Results of the COCHE study. Haematologica.

[18] Mccusker P.J., Fischer K., Holzhauer S., Meunier S., Altisent C., Grainger J.D. (2015). International cross-cultural validation study of the Canadian haemophilia outcomes: kids’ life assessment tool. Haemophilia.

[19] Young N.L., Bradley C.S., Wakefield C.D., Barnard D., Blanchette V.S., McCusker P.J. (2006). How well does the Canadian Haemophilia Outcomes-Kids’ Life Assessment Tool (CHO-KLAT) measure the quality of life of boys with haemophilia?. Pediatr Blood Cancer.

[20] von Mackensen S., Gringeri A., Preedy V., Watson R. (2010).

[21] Furnée C.A., Groot W., Van Den Brink H.M. (2008). The health effects of education: a meta-analysis. Eur J Public Health.

[22] Stat Netherlands Practical and theoretical education. https://www.cbs.nl/nl-nl/longread/discussion-papers/2021/invulling-praktisch-en-theoretisch-opgeleiden/3-indeling-van-opleidingen-op-basis-van-niveau-en-orientatie.

[23] van Balen E.C., Hassan S., Smit C., Driessens M.H.E., Beckers E.A.M., Coppens M. (2022). Socioeconomic participation of persons with hemophilia: results from the sixth hemophilia in the Netherlands study. Res Pract Thromb Haemost.

[24] Haverman L., Engelen V., van Rossum M.A.J., Heymans H.S.A., Grootenhuis M.A. (2011). Monitoring health-related quality of life in paediatric practice: development of an innovative web-based application. BMC Pediatr.

[25] Riske B., Shearer R., Baker J.R. (2020). Patient satisfaction with US Hemophilia Treatment Center Care, Teams and Services: the First National Survey. Haemophilia.

[26] von Mackensen S., Schleicher C., Heine S., Graf N., Eichler H. (2020). Health-related quality of life, treatment satisfaction and adherence outcomes of haemophilia patients living in a German rural region. Hamostaseologie.

[27] van Balen E.C., Wesselo M.L., Baker B.L., Westerman M.J., Coppens M., Smit C. (2020). Patient perspectives on novel treatments in haemophilia: a qualitative study. Patient.

[28] von Mackensen S., Kalnins W., Krucker J., Weiss J., Miesbach W., Albisetti M. (2017). Haemophilia patients’ unmet needs and their expectations of the new extended half-life factor concentrates. Haemophilia.

[29] McLenon J., Rogers M.A.M. (2019). The fear of needles: a systematic review and meta-analysis. J Adv Nurs.

[30] Lane S.J., Sholapur N.S., Yeung C.H.T., Iorio A., Heddle N.M., Sholzberg M. (2016). Understanding stakeholder important outcomes and perceptions of equity, acceptability and feasibility of a care model for haemophilia management in the US: a qualitative study. Haemophilia.

[31] Federation of Medical Specialists, CMIO Network (2019). Medical data exchange (Gegevensuitwisseling). https://www.demedischspecialist.nl/onderwerp/gegevensuitwisseling.

[32] Devine E.B., Totten A.M., Gorman P., Eden K.B., Kassakian S., Woods S. (2017). Health Information Exchange Use (1990-2015): a systematic review. eGEMs (Wash DC).

[33] Urden L.D. (2002). Patient satisfaction measurement: current issues and implications. Lippincotts Case Manag.

