# Supplementary material for: Patients’ and health care providers’ perspectives on quality of hemophilia care in the Netherlands: a questionnaire and interview study
Source: Res Pract Thromb Haemost. 2023 Apr 23;7(4):100159. doi: 10.1016/j.rpth.2023.100159 (PMC10279896; doi:10.1016/j.rpth.2023.100159)
Supplement: Supplemental Tables 1 and 2 [file mmc1.docx]

**Appendices**

**Table S1.** **Hemo-Sat questionnaire standardized scores**

|  | No. of items | Adults, median (IQR) | n | Parents, median (IQR) | n |
| --- | --- | --- | --- | --- | --- |
| Ease and convenience | 10 (11*) | 17.5 (7.5-30.0) | 609 | 22.5 (9.1-32.3) | 108 |
| Efficacy | 6 | 16.7 (8.3-33.3) | 598 | 20.8 (8.3-33.3) | 108 |
| Burden | 4 | 12.5 (0-25.0) | 624 | 29.4 (11.8-41.2) | 114 |
| Specialist/nurses | 7 | 0 (0-7.1) | 638 | 0 (0-12.5) | 117 |
| Center/hospital | 5 | 0 (0-6.3) | 650 | 0 (0-6.3) | 119 |
| General satisfaction | 2 | 0 (0-12.5) | 649 | 0 (0-12.5) | 111 |
| Total | 34 (35*) | 11.8 (5.1-19.9) | 593 | 15.0 (7.4-22.9) | 111 |

*In the Hemo-SatP questionnaire for parents, there was 1 additional item in the ‘Ease & convenience’ domain, adding up to 11 items in that domain and 35 items in total.

**Table S2.** **Hemo-Sat questionnaire standardized scores, divided by participant characteristics**

| **A Prophylaxis use** | **Adults** |  |  |  |  |  |  | **Parents of children aged < 12 years** | | | | | | |
| --- | --- | --- | --- | --- | --- | --- | --- | --- | --- | --- | --- | --- | --- | --- |
| Hemo-Sat domain | Using prophylaxis, median (IQR) | n | On demand, median (IQR) | n | Diff^*^ | Assumed MID^#^ | p-value | Using prophylaxis, median (IQR) | n | On demand median (IQR) | n | Diff^*^ | Assumed MID^#^ | p-value |
| Ease/convenience | 12.5 (5.0-25.0) | 234 | 20.0 (10.0-32.5) | 375 | 7.5 | 7.4 | 0.00 | 13.6 (6.8-27.3) | 61 | 27.3 (18.2-36.4) | 47 | 13.7 | 7.2 | 0.00 |
| Efficacy | 16.7 (4.2-29.2) | 227 | 16.7 (8.3-33.3) | 370 | 0 | 9.1 | NS | 16.7 (0-29.2) | 60 | 22.9 (8.3-33.3) | 48 | 6.2 | 7.8 | NS |
| Burden | 12.5 (0-31.3) | 228 | 12.5 (0-25.0) | 395 | 0 | 9.0 | NS | 17.6 (5.9-35.3) | 63 | 35.3 (23.5-41.2) | 51 | 17.7 | 8.9 | 0.00 |
| Specialist/nurses | 0 (0-10.7) | 226 | 0 (0-7.1) | 411 | 0 | 6.6 | NS | 3.6 (0-14.3) | 61 | 0 (0-7.1) | 56 | 3.6 | 5.3 | NS |
| Center/hospital | 0 (0-10.0) | 225 | 0 (0-5.0) | 424 | 0 | 6.3 | NS | 0 (0-8.2) | 61 | 0 (0-6.3) | 58 | 0 | 5.7 | NS |
| General satisfaction | 0 (0-12.5) | 225 | 0 (0-12.5) | 423 | 0 | 7.6 | NS | 0 (0-12.5) | 60 | 0 (0-12.5) | 51 | 0 | 9.3 | NS |
| Total score | 10.3 (4.4-19.9) | 222 | 11.8 (5.9-19.9) | 371 | 1.5 | 5.9 | NS | 12.9 (5.0-22.1) | 61 | 17.1 (11.7-23.8) | 50 | 4.2 | 5.0 | NS |

| **B Disease severity** | **Adults** |  |  |  |  |  |  | **Parents of children aged < 12 years** | | | | | | |
| --- | --- | --- | --- | --- | --- | --- | --- | --- | --- | --- | --- | --- | --- | --- |
| Hemo-Sat domain | Severe hemophilia, median (IQR) | n | Non-severe hemophilia, median (IQR) | n | Diff^*^ | Assumed MID^#^ | p-value | Severe hemophilia, median (IQR) | n | Non-severe hemophilia, median (IQR) | n | Diff^*^ | Assumed MID^#^ | p-value |
| Ease/convenience | 11.1 (2.5-25.0) | 242 | 20.0 (10.0-35.0) | 368 | 8.9 | 7.4 | 0.00 | 11.4 (6.8-27.3) | 55 | 27.3 (18.2-36.4) | 53 | 15.9 | 7.2 | 0.00 |
| Efficacy | 16.7 (5.0-33.3) | 235 | 16.7 (8.3-33.3) | 363 | 0 | 9.1 | NS | 14.6 (0-30.2) | 54 | 20.8 (8.3-33.3) | 54 | 6.2 | 7.8 | NS |
| Burden | 12.5 (0-25.0) | 238 | 12.5 (0-25.0) | 387 | 0 | 9.0 | NS | 17.6 (5.9-38.2) | 57 | 30.1 (23.5-41.2) | 57 | 12.5 | 8.9 | 0.00 |
| Specialist/nurses | 0 (0-10.7) | 235 | 0 (0-7.1) | 404 | 0 | 6.6 | NS | 3.6 (0-14.3) | 55 | 0 (0-10.7) | 62 | 3.6 | 5.3 | NS |
| Center/hospital | 0 (0-10.0) | 233 | 0 (0-5.0) | 418 | 0 | 6.3 | NS | 0 (0-10.0) | 55 | 0 (0-5.0) | 64 | 0 | 5.7 | NS |
| General satisfaction | 0 (0-12.5) | 233 | 0 (0-12.5) | 417 | 0 | 7.6 | NS | 0 (0-12.5) | 54 | 0 (0-25.0) | 57 | 0 | 9.3 | NS |
| Total score | 10.3 (4.4-19.9) | 230 | 11.8 (5.9-19.9) | 364 | 1.5 | 5.9 | NS | 12.9 (4.3-22.1) | 55 | 17.1 (10.5-24.1) | 56 | 4.2 | 5.0 | 0.02 |

*Difference in median scores

#Since the minimal important difference (MID) has not been determined for the Hemo-Sat questionnaire, a widely accepted approximation of the MID is used: half a standard deviation. The MID indicates the smallest difference which would still be of clinical value to patients. If the difference in median scores is higher than the (assumed) MID, this difference is likely of clinical importance.

^p-value

**The median (IQR) scores of adults with an eradicated inhibitor and adults who never had an inhibitor were nearly identical. Therefore, the scores of adults with a current inhibitor and adults who never had an inhibitor are compared. Since only one parent of a child with a current inhibitor completed all Hemo-SAT domains, we compared the scores of parents of children with an eradicated inhibitor to the scores of parents of a child who never had an inhibitor.

Abbreviations: IQR, interquartile range; MID, minimal important difference; NS, not significant
